# High-Throughput Assessment of Drug Cardiac Safety Using a High-Speed Impedance Detection Technology-Based Heart-on-a-Chip

**DOI:** 10.3390/mi7070122

**Published:** 2016-07-19

**Authors:** Xi Zhang, Tianxing Wang, Ping Wang, Ning Hu

**Affiliations:** 1 Key Laboratory of Biomedical Engineering of Ministry of Education, Biosensor National Special Laboratory, Department of Biomedical Engineering, Zhejiang University, Hangzhou 310027, China; xi.zhang1000@gmail.com (X.Z.); frankwang@zju.edu.cn (T.W.); cnpwang@zju.edu.cn (P.W.); 2 ACEA Biosciences (Hangzhou) Co., Ltd., High Throughput Drug Screening Center, Xihu Technology and Economy Park, Hangzhou 310030, China; 3 Division of Biomedical Engineering, Biomaterials Innovation Research Center, Department of Medicine, Brigham and Women’s Hospital, Harvard Medical School, Cambridge, MA 02139, USA; 4 Harvard-MIT Health Sciences and Technology, Massachusetts Institute of Technology, Cambridge, MA 02139, USA

**Keywords:** heart-on-a-chip, impedance detection technology, drug cardiac efficacy, high throughput assessment

## Abstract

Drug cardiac safety assessments play a significant role in drug discovery. Drug-induced cardiotoxicity is one of the main reasons for drug attrition, even when antiarrhythmic drugs can otherwise effectively treat the arrhythmias. Consequently, efficient drug preclinical assessments are needed in the drug industry. However, most drug efficacy assessments are performed based on electrophysiological tests of cardiomyocytes in vitro and cannot effectively provide information on drug-induced dysfunction of cardiomyocyte beating. Here we present a heart-on-a-chip device for evaluating the drug cardiac efficacy using a high-speed impedance detection technology. Verapamil and doxorubicin were utilized to test this heart-on-a-chip, and multiple parameters of cardiomyocyte beating status are used to reveal the effects of drugs. The results show that drug efficacy or cardiotoxicity can be determined by this heart-on-a-chip. We believe this heart-on-a-chip will be a promising tool for the preclinical assessment of drug cardiac efficacy.

## 1. Introduction

Drug cardiac safety determination is one of the most important processes in drug discovery and development. Drug safety includes drug-induced cardiotoxicity and drug efficacy assessment. It is challenging to assess drug efficacy and drug side effects at an early stage by effectively selecting the most promising candidate drugs and decreasing their safety-related attrition [1,2]. Drug-induced cardiotoxicity accounts for a large fraction of safety-related drug attrition [2,3]. Consequently, there is an urgent need to identify suitable methods to assess drug cardiac safety. Preclinical cardiotoxicity screening in vitro based on heart-on-a-chip technologies provides an efficient manner of predicting unsafe compounds by monitoring the electrophysiological and mechanical properties of cardiomyocytes.

The patch clamp serves as the gold standard for detection of electrophysiological properties in order to assess drug effects on ion channels [4,5]. Many label-based methods have emerged by monitoring action potential or calcium transient. However, invasive or label-based low-throughput detection methods will preclude large-scale monitoring and long-term drug testing due to the adverse effects of these technologies. Microelectrode array (MEA) provides an alternative approach to test the effect of drugs by recording the extracellular potential of cardiomyocytes in a non-invasive, high-throughput, and long-term way [6,7,8].

The methods of detecting electrophysiological properties can essentially evaluate drug effects on ion channels of cardiomyocytes. Nevertheless, they cannot monitor their mechanical contraction status, which is responsibile for pumping blood from the heart to other organs. Mechanical contraction can be interrupted by other component inhibition on myofilaments, and these drug effects cannot be assessed by electrophysiological detection methods [9,10,11]. Therefore, it is necessary to establish a platform for mechanical contraction of cardiomyocytes. There are many detection methods for mechanical contraction, such as soft cantilever [12,13] and micropillar [14,15]. These heart-on-a-chip technologies can directly assess the mechanical contraction status. However, contraction was influenced by large counterforces within these structures. Hence, other mechanical contraction detection methods are needed to monitor the native contraction status. Cardiomyocytes can rhythmically contract and relax on the chamber surface, and the surface-cardiomyocyte interface will also rhythmically change, so these contraction behaviors can be detected by monitoring the surface alternations. Impedance detection technology is a utility method for attachment status which was firstly proposed to detect the cell attachment and morphology [16,17]. However, the conventional impedance technology was applied for analyzing cell growth with a low sampling rate, which makes it difficult to monitor rapid impedance fluctuation such as cardiomyocyte beating.

In this work, we reported a high-speed impedance detection technology-based heart-on-a-chip device using primary neonatal rat cardiomyocytes as the research model. The performance of the heart-on-a-chip device was tested by typical antiarrhythmic and anticancer drugs. The beating rate and amplitude were derived from the mechanical contraction signals, which were well-matched with the drug effects. We believe this heart-on-a-chip will be a promising platform for drug cardiotoxicity in drug discovery.

## 2. Results

### 2.1. Establishment of Heart-on-a-Chip

**Figure 1:**
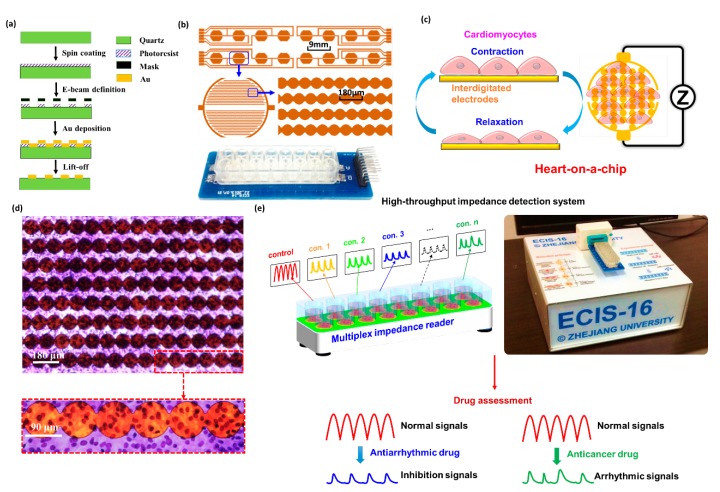
Establishment of heart-on-a-chip for drug assessment. (**a**) The fabrication of interdigitated electrodes (IDEs); (**b**) The IDEs layout and image of device; (**c**) Schematics of heart-on-a-chip with IDEs and impedance detection method; (**d**) The Diff-Quik staining images of cardiomyocytes on the device; (**e**) High-throughput impedance detection system for drug assessment using beating characteristics.

To establish the heart-on-a-chip device, interdigitated electrodes (IDEs) and primary neonatal rat cardiomyocytes were used. IDEs were fabricated by electron beam lithography, Au deposition, and lift-off protocol (Figure 1a). Each device contained 16 IDEs with 5 mm in diameter, 9 mm in center-to-center spacing, and the distance between adjacent IDEs branches was about 110 μm. For the purpose of cell culture, a polystyrene chamber was fixed on the defined IDEs pattern with epoxy glue to establish the cell culture chamber (Figure 1b).

The cardiomyocytes rhythmically contracted and relaxed after they were cultured on IDEs for 1–2 days, and the alternations of cardiomyocyte attachment status would induce the IDEs impedance changes (Figure 1c). To determine the cell distribution on the IDEs, the primary rat cardiomyocytes were labeled by Diff-Quik staining, an improved Romanowsky staining method [18]. It can clearly be seen that the primary rat cardiomyocytes are uniformly distributed on the device surface with violet nucleus and light blue cytoplasm (Figure 1d).

The impedance fluctuation induced by the contraction and relaxation of cardiomyocytes can be measured by the high-speed impedance detection technology in real time (Figure 1e). To normalize impedance signals, cell index (CI) was introduced, which was defined as the ratio of impedance change and background impedance of IDEs. After the heart-on-a-chip was fabricated, drug assessment was performed when the beating signals became stable. The beating characteristics, such as beating rate and amplitude, can easily reflect the drug effects. Moreover, the cardiotoxicity can be determined by arrhythmic beating profile.

### 2.2. Heart-on-a-Chip for Antiarrhythmic Drug Test

**Figure 2:**
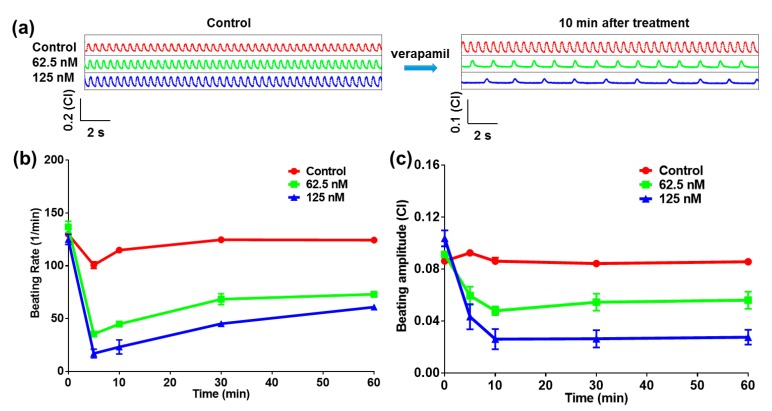
Heart-on-a-chip for verapamil test. (**a**) The beating signals before and after the verapamil with different concentrations are administered on the cardiomyocytes; (**b**) Statistical beating rate within 60 min in the presence of verapamil; (**c**) Statistical beating rate within 60 min in the presence of verapamil.

To evaluate the drug efficacy test function of the heart-on-a-chip device, verapamil, which is a class IV antiarrhythmic drug and used as the L-type calcium channel blocker, was applied to test the performance of the heart-on-a-chip device. Verapamil would reduce the calcium influx together with the action potential, leading to decreases of both the beating rate and contractility [19]. For verapamil effect assessment, verapamil solutions with different concentrations (62.5 nM, 125 nM) were tested. When cardiomyocytes were cultured for 40 h and cells beat rhythmically with the same rate, the verapamil solutions were added into the wells. Figure 2a showed that the beating status of the cardiomyocytes significantly changed after treatment for 10 min. It was obvious that the beating rates of cardiomyocytes decreased from 114.9 ± 2.1 (Control) to 44.9 ± 1.1 (62.5 nM, *p* = 0.0011, *n* = 3) and 23.2 ± 6.6 (125 nM, *p* = 0.0057, *n* = 3) beats/min after 10 min drug treatment. Meanwhile, the beating amplitudes of cardiomyocytes decreased from 0.0862 ± 0.003 (Control) to 0.0480 ± 0.0033 (62.5 nM, *p* = 0.0122, *n* = 3) and 0.0262 ± 0.008 (125 nM, *p* = 0.0182, *n* = 3) (CI). Verapamil would inhibit the inflow of calcium ion during the phase II of the cardiomyocytes action potential. A larger number of free calcium ions were inhibited from entering into the cells, and less calcium would be released from the intracellular calcium source. Finally, the contractility of the cardiomyocytes decreased, which was matched with a decrease in the cardiomyocyte beating parameters [20]. The beating rate curves also showed dose and time dependent recovery in the long term. However, the beating rates of the treatment groups were less than those of the control group. For the further investigation of verapamil effects, both the beating rate and the beating amplitude were extracted from the raw data. From statistical results in Figure 2b,c, the chronotropic effects could be observed, which were both well-matched with the verapamil effect. It was demonstrated that this heart-on-a-chip could accurately reflect the drug efficacy.

### 2.3. Heart-on-a-Chip for Anticancer Drug Test

**Figure 3:**
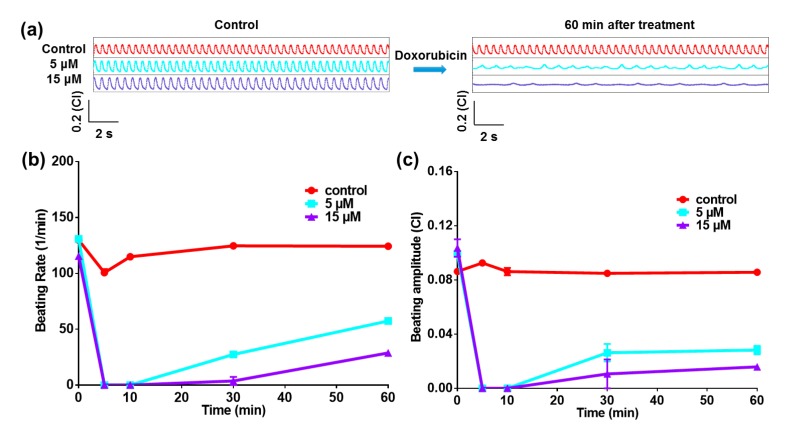
Heart-on-a-chip for doxorubicin test. (**a**) The beating signals before and after the doxorubicin with different concentrations are administered on the cardiomyocytes; (**b**) Statistical beating rate within 60 min in the presence of doxorubicin; (**c**) Statistical beating rate within 60 min in the presence of doxorubicin.

To evaluate the drug-induced cardiotoxicity test function of the heart-on-a-chip device, a typical anticancer drug, doxorubicin, was applied to test the performance of the heart-on-a-chip device. As shown in Figure 3a, the beating rate and amplitude were both significantly reduced after adding the doxorubicin into chips. It was obvious that the beating rates of cardiomyocytes decreased from 124.3 ± 0.7 (Control) to 57.4 ± 1.4 (5 μM, *p* = 0.0005, *n* = 3) and 28.8 ± 2.9 (15 μM, *p* = 0.0010, *n* = 3) beats/min after 60 min drug treatment. Meanwhile, the beating amplitudes of cardiomyocytes decreased from 0.0857 ± 0.001 (Control) to 0.0282 ± 0.003 (5 μM, *p* = 0.0033, *n* = 3) and 0.0159 ± 0.001 (15 μM, *p* = 0.0003, *n* = 3) (CI). Besides, the arrhythmic beating signals appeared after treatment of 60 min. For the further investigation of doxorubicin effects, both the beating rate and beating amplitude were extracted from the raw data. From the statistical results in Figure 3b,c, the chronotropic effects could be observed. Doxorubicin is one kind of anthracycline anticancer drug, whose dangerous adverse effect is cardiomyopathy, resulting in the cardiomyocyte apoptosis and congestive heart failure [21]. There are several ways in which doxorubicin is believed to cause cardiomyopathy, including oxidative stress, downregulation of genes for contractile proteins, and p53 mediated apoptosis [22]. The cardiotoxicity of doxorubicin could be reflected by the arrhythmic profile of beating signals. It was demonstrated that this heart-on-a-chip could accurately reflect the drug that was induced by the beating pattern functions.

### 2.4. Cell Viability Test by Impedance Detection Method

**Figure 4:**
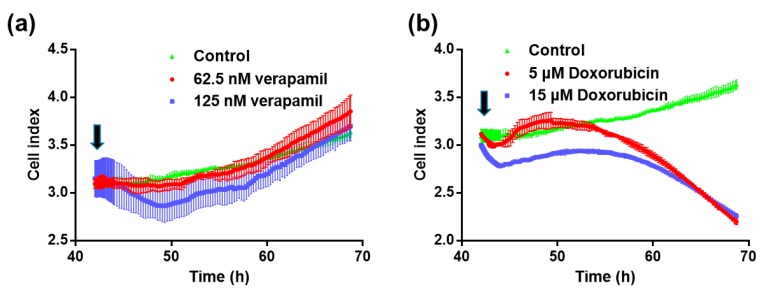
Cell viability test by the impedance detection method. (**a**) Cell growth curves in the absence and presence of verapamil; (**b**) Cell growth curves in the absence and presence of doxorubicin. Black arrows show the initial time of drug treatment.

In former research, it has been proved that the cell growth curve can reflect the cell viability [23]. The cell growth curves had similar results to the cell counting Kit-8 assay (CCK8). Cell number, morphology, and attachment on the IDEs would affect the impedance (cell index values), which was related to the cell viability. Consequently, the cell growth curves were used to indicate cell viability in a label-free way. As shown in Figure 4, cell growth curves of two drugs had different trends due to the cardiotoxicity of drugs. Compared with the control group, the cell index still increased with the verapamil treatment, while it significantly decreased with the doxorubicin treatment.

**Figure 5:**
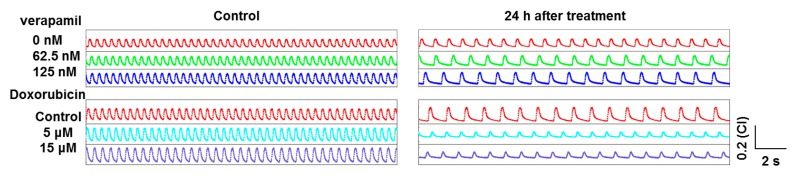
Long-term monitoring (24 h) after drug treatment.

Based on the label-free and noninvasive impedance technology, the beating status of cardiomyocytes can also be monitored in a long-term way. Figure 5 shows the typical signals of these two drugs after 24 h treatment. Compared with the control group and former short-term signals, the beating signals recovered to the normal status in the presence of verapamil after 24 h. The beating signals become rhythmic in the presence of doxorubicin after 24 h. However, the amplitudes of the beating signals were smaller than in the control group due to cardiotoxicity.

## 3. Materials and Methods

### 3.1. Device Fabrication

The device was fabricated on a non-conductive substrate with a gold pattern. Initially, a four-inch quartz wafer was cut into 80 mm × 20 mm pieces, and each piece was defined by interdigitated electrodes arranged in an eight-by-two grid, and their lead using electron beam lithography, 100 nm Au deposition and liftoff processes. An eight-by-two polystyrene chamber was fixed on the defined IDEs pattern with epoxy glue to insulate the leads outside the chamber. The leads of device were connected to external circuits by a printed circuit board.

### 3.2. Cell Culture

One-day-old rats were sterilized by 75% alcohol, and the chest wall of the rat was cut by scissors. The heart was rapidly isolated from the neonatal rat, which was rinsed in iced Dulbecco’s modified Eagle medium (DMEM) and washed to remove blood and debris. Atriums were removed, while ventricle tissues were left and moved into 2 mL Hanks balanced salt solution (HBSS). Subsequently, ventricles were shredded into 1 mm^3^ tissue fragments. These fragments were digested by stepwise trypsin and collagenase II for 10 times after HBSS removal. The fragments were slowly insulated using a glass pipette several times to make the cell dissociated. Supernatant was removed and DMEM with 10% *v*/*v* Fetal Bovine Serum (FBS) was used to stop digestion. Cell suspension was centrifuged with 800 rpm for 5 min. The suspension was removed and 4 mL DMEM with 10% *v*/*v* FBS was added to resuspend the cells. Finally, 17,000 cells were cultured in each chamber of the device, which was coated with 0.1% *w*/*v* gelatin in 4 °C refrigerator overnight. The device was fixed on the detection system in a humidified 37 °C, 5% CO_2_ incubator. The medium was changed every 24 h. The drug experiments were usually performed after 40 h cell culture. To determine the distribution on the device, Diff-Quik staining (Siemens), an improved Romanowsky staining, was used to label the primary neonatal rat cardiomyocytes.

### 3.3. Impedance Measurement

For the impedance measurement, sine voltage drive signals (10 kHz in frequency and 30 mV in amplitude) were applied onto one of the IDEs. The ion current will flow out from the other one. To detect the sine current, the signals should be converted and amplified (6000×) into the voltage signals with suitable amplitudes for data acquisition. A 1 M/s sampling rate was used to obtain the amplified sine voltage signals, and the amplitude of sine voltage signals was derived by fast Fourier transform. The impedance can be calculated based on the drive signals and detected voltage signals. The final sampling rate for beating signals was about 500 Hz.

### 3.4. Drug Treatment

To test the performance of this heart-on-a-chip device, two typical tool drugs (verapamil and doxorubicin) were selected. The concentration of verapamil and doxorubicin were chosen based on the former study [24,25]. The highest concentration of verapamil was 2 μM, and then it was diluted into 1 μM, 0.5 μM, 0.25 μM, 0.125 μM, and 0.0625 μM, and we performed the drug experiments separately. The highest concentration of doxorubicin was 15 μM, and then it was diluted into 5 μM, 1.67 μM, 0.56 μM, 0.19 μM, and 0.062 μM, and we performed the drug experiments separately.

### 3.5. Statistical Analysis

All results were presented as mean and standard deviation. Data were performed with an unpaired Student’s *t*-test using Graphpad Prism 6. *p* < 0.05 was considered statistically significant. 

## 4. Discussion and Conclusions

The conventional impedance technology has a low sampling rate (e.g., 1 Hz), which is only able to monitor the growth behavior of cardiomyocytes [16,17]. However, this low-speed impedance detection technology cannot be applied in rapid response cardiomyocyte beating. To record the rapid behaviors of cardiomyocyte beating, the sampling rate should be higher. In the study, we use the high sampling rate (500 Hz) to record the rapid impedance fluctuations induced by the cardiomyocyte beating. Moreover, the high-speed impedance technology can also record the cell growth status.

Other successful heart-on-a-chip platforms have been reported for cardiomyocyte contraction study by the polydimethylsiloxane (PDMS)-based microcantilever or micropilliar [12,13,14,15,26]. However, the force from the PDMS will affect the native contraction of cardiomyocytes, and the Conserved Domain Database (CDD) recording was demanded in the experiments. Compared with these platforms, our impedance technology-based heart-on-a-chip platform can record the contraction of cardiomyocyte in a native way. Moreover, the phototoxicity can be totally avoided. Therefore, the heart-on-a-chip device has unique advantages in drug assessment.

The heart-on-a-chip device can determine the drug efficacy and cardiotoxicity by typical antiarrhythmic and anticancer drugs tests. Compared with the action potential and extracellular potential recorded by the electrophysiological methods, the beating signals recorded by impedance detection method can directly assess the drug effects by mechanical contraction status, which was directly related to the pumping blood function of the heart. Consequently, the monitoring of mechanical contraction status of cardiomyocytes plays an important role in drug assessment. In previous research, we focused on the comparisons of cardiomyocyte beating detection by CCD imaging and impedance methods, while the ion channel tool drugs were employed to increase and decrease the contractility of cardiomyocyte beating [27]. Here we introduced the beating pattern function for the drug-induced cardiotoxicity by an anticancer drug, doxorubicin. Moreover, the cell viability can be determined by the cell growth curves. Therefore, this multifunctional heart-on-a-chip device will potentially provide a label-free and noninvasive platform for preclinical drug assessment.

In the next step, the microelectrode or nanoelectrode array can be integrated with interdigitated electrodes, and thus the potential effects and contraction signals of cardiomyocyte can be simultaneously recorded, which allow us to fully obtain the information regarding drug effect from both characteristics. Furthermore, the excitation-contraction behaviors of cardiomyocyte can be monitored by the integrated device to establish a new heart-on-a-chip for drug discovery or precision medicine.

## References

[1] Gintant G., Sager P.T., Stockbridge N. (2016). Evolution of strategies to improve preclinical cardiac safety testing. Nat. Rev. Drug Discov..

[2] Bowes J., Brown A.J., Hamon J., Jarolimek W., Sridhar A., Waldron G., Whitebread S. (2012). Reducing safety-related drug attrition: The use of in vitro pharmacological profiling. Nat. Rev. Drug Discov..

[3] Kola I., Landis J. (2004). Can the pharmaceutical industry reduce attrition rates?. Nat. Rev. Drug Discov..

[4] Hamill O.P., Marty A., Neher E., Sakmann B., Sigworth F. (1981). Improved patch-clamp techniques for high-resolution current recording from cells and cell-free membrane patches. Pflügers Archiv..

[5] Sakmann B., Neher E. (2009). Single-Channel Recording.

[6] Hu N., Fang J., Li H., Su K., Wang P. Dual-Function Microelectrode Array System for Simultaneously Monitoring Electromechanical Integration Status of Cardiomyocytes. Proceedings of the 18th International Conference on Solid-State Sensors, Actuators and Microsystems (Transducers '15).

[7] Navarrete E.G., Liang P., Lan F., Sanchez-Freire V., Simmons C., Gong T., Sharma A., Burridge P.W., Patlolla B., Lee A.S. (2013). Screening Drug-Induced Arrhythmia Using Human Induced Pluripotent Stem Cell–Derived Cardiomyocytes and Low-Impedance Microelectrode Arrays. Circulation.

[8] Liu Q., Wu C., Cai H., Hu N., Zhou J., Wang P. (2014). Cell-based biosensors and their application in biomedicine. Chem. Rev..

[9] Dou Y., Arlock P., Arner A. (2007). Blebbistatin specifically inhibits actin-myosin interaction in mouse cardiac muscle. Am. J. Physiol. Cell Physiol..

[10] Fedorov V.V., Lozinsky I.T., Sosunov E.A., Anyukhovsky E.P., Rosen M.R., Balke C.W., Efimov I.R. (2007). Application of blebbistatin as an excitation–contraction uncoupler for electrophysiologic study of rat and rabbit hearts. Heart Rhythm.

[11] Kovács M., Tóth J., Hetényi C., Málnási-Csizmadia A., Sellers J.R. (2004). Mechanism of blebbistatin inhibition of myosin II. J. Biol. Chem..

[12] Agarwal A., Goss J.A., Cho A., McCain M.L., Parker K.K. (2013). Microfluidic heart on a chip for higher throughput pharmacological studies. Lab Chip.

[13] Grosberg A., Alford P.W., McCain M.L., Parker K.K. (2011). Ensembles of engineered cardiac tissues for physiological and pharmacological study: Heart on a chip. Lab Chip.

[14] Tanaka Y., Morishima K., Shimizu T., Kikuchi A., Yamato M., Okano T., Kitamori T. (2006). Demonstration of a PDMS-based bio-microactuator using cultured cardiomyocytes to drive polymer micropillars. Lab Chip.

[15] Morishima K., Tanaka Y., Ebara M., Shimizu T., Kikuchi A., Yamato M., Okano T., Kitamori T. (2006). Demonstration of a bio-microactuator powered by cultured cardiomyocytes coupled to hydrogel micropillars. Sens. Actuators B Chem..

[16] Giaever I., Keese C. (1984). Monitoring fibroblast behavior in tissue culture with an applied electric field. Proc. Natl. Acad. Sci. USA.

[17] Giaever I., Keese C.R. (1993). A morphological biosensor for mammalian cells. Nature.

[18] Enginsu M., Dumoulin J., Pieters M., Bras M., Evers J., Geraedts J. (1991). Evaluation of human sperm morphology using strict criteria after Diff-Quik staining: Correlation of morphology with fertilization in vitro. Hum. Reprod..

[19] Oudit G.Y., Sun H., Trivieri M.G., Koch S.E., Dawood F., Ackerley C., Yazdanpanah M., Wilson G.J., Schwartz A., Liu P.P. (2003). L-type Ca^2+^ channels provide a major pathway for iron entry into cardiomyocytes in iron-overload cardiomyopathy. Nat. Med..

[20] Bers D.M. (2002). Cardiac excitation–contraction coupling. Nature.

[21] Arola O.J., Saraste A., Pulkki K., Kallajoki M., Parvinen M., Voipio-Pulkki L.-M. (2000). Acute doxorubicin cardiotoxicity involves cardiomyocyte apoptosis. Cancer Res..

[22] Chatterjee K., Zhang J., Honbo N., Karliner J.S. (2010). Doxorubicin cardiomyopathy. Cardiology.

[23] Zou L., Wang Q., Tong M., Li H., Wang J., Hu N., Wang P. (2016). Detection of diarrhetic shellfish poisoning toxins using high-sensitivity human cancer cell-based impedance biosensor. Sens. Actuators B Chem..

[24] Kimura S., Bassett A.L., Xi H., Myerburg R.J. (1992). Verapamil diminishes action potential changes during metabolic inhibition by blocking ATP-regulated potassium currents. Circ. Res..

[25] Xiao L., Hu Z., Zhang W., Wu C., Yu H., Wang P. (2010). Evaluation of doxorubicin toxicity on cardiomyocytes using a dual functional extracellular biochip. Biosens. Bioelectron..

[26] Wang G., McCain M.L., Yang L., He A., Pasqualini F.S., Agarwal A., Yuan H., Jiang D., Zhang D., Zangi L. (2014). Modeling the mitochondrial cardiomyopathy of Barth syndrome with induced pluripotent stem cell and heart-on-chip technologies. Nat. Med..

[27] Su K., Zou Q., Li H., Wang T., Zhuang L., Hu N., Wang P. (2015). Cardiomyocyte-Based Biosensor Based on Impedance Sensor Technology and CCD Imaging Analysis for Pharmaceutical Assessment. Sens. Lett..

